# Supplementary material for: Human intraparietal sulcal morphology relates to individual differences in language and memory performance
Source: Commun Biol. 2024 May 2;7:520. doi: 10.1038/s42003-024-06175-9 (PMC11065983; doi:10.1038/s42003-024-06175-9)
Supplement: Supplementary file 2 — Description of Additional Supplementary Files [file 42003_2024_6175_MOESM2_ESM.pdf]

Description of Additional Supplementary Files

File name: Supplementary Data 1
Description: Source data underlying Figure 2

File name: Supplementary Data 2
Description: Source data underlying Figure 3

File name: Supplementary Data 3
Description: Source data underlying Figure 4
