# Supplementary material for: Human intraparietal sulcal morphology relates to individual differences in language and memory performance
Source: Commun Biol. 2024 May 2;7:520. doi: 10.1038/s42003-024-06175-9 (PMC11065983; doi:10.1038/s42003-024-06175-9)
Supplement: Supplementary file 6 — Reporting Summary [file 42003_2024_6175_MOESM6_ESM.pdf]

nature portfolio

Corresponding author(s): Giorgia Committeri and Federica Santacroce

Last updated by author(s): Feb 22, 2024

Reporting Summary

Nature Portfolio wishes to improve the reproducibility of the work that we publish. This form provides structure for consistency and transparency in reporting. For further information on Nature Portfolio policies, see our Editorial Policies and the Editorial Policy Checklist.

Statistics

For all statistical analyses, confirm that the following items are present in the figure legend, table legend, main text, or Methods section.

n/a	Confirmed	
☐	☒	The exact sample size (n) for each experimental group/condition, given as a discrete number and unit of measurement
☒	☐	A statement on whether measurements were taken from distinct samples or whether the same sample was measured repeatedly
☐	☒	The statistical test(s) used AND whether they are one- or two-sided<br>*Only common tests should be described solely by name; describe more complex techniques in the Methods section.*
☐	☒	A description of all covariates tested
☒	☐	A description of any assumptions or corrections, such as tests of normality and adjustment for multiple comparisons
☐	☒	A full description of the statistical parameters including central tendency (e.g. means) or other basic estimates (e.g. regression coefficient) AND variation (e.g. standard deviation) or associated estimates of uncertainty (e.g. confidence intervals)
☐	☒	For null hypothesis testing, the test statistic (e.g. F, t, r) with confidence intervals, effect sizes, degrees of freedom and P value noted<br>*Give P values as exact values whenever suitable.*
☒	☐	For Bayesian analysis, information on the choice of priors and Markov chain Monte Carlo settings
☒	☐	For hierarchical and complex designs, identification of the appropriate level for tests and full reporting of outcomes
☐	☒	Estimates of effect sizes (e.g. Cohen's d, Pearson's r), indicating how they were calculated

Our web collection on statistics for biologists contains articles on many of the points above.

Software and code

Policy information about availability of computer code

Data collection	Workbench (wb_view) for sulcal labelling.
Data analysis	IBM SPSS Statistic 25

For manuscripts utilizing custom algorithms or software that are central to the research but not yet described in published literature, software must be made available to editors and reviewers. We strongly encourage code deposition in a community repository (e.g. GitHub). See the Nature Portfolio guidelines for submitting code & software for further information.

Data

Policy information about availability of data

All manuscripts must include a data availability statement. This statement should provide the following information, where applicable:

- Accession codes, unique identifiers, or web links for publicly available datasets
- A description of any restrictions on data availability
- For clinical datasets or third party data, please ensure that the statement adheres to our policy

No experimental datasets were collected in this study. The MRI and behavioural datasets are freely available from the Human Connectome Project (HPC) website, 1200 Subjects Release (S1200) at this address: https://db.humanconnectome.org/app/template/Login.vm.

Research involving human participants, their data, or biological material

Policy information about studies with human participants or human data. See also policy information about sex, gender (identity/presentation), and sexual orientation and race, ethnicity and racism.

Reporting on sex and gender	All information are available from the freely available Human Connectome Project (HPC) website, 1200 Subjects Release (S1200) at this address: https://db.humanconnectome.org/app/template/Login.vm.
Reporting on race, ethnicity, or other socially relevant groupings	No information on race, ethnicity or other social groupings was used in the study.
Population characteristics	Age range of the healthy sample: 26-30 years.
Recruitment	See above (use of a freely available dataset).
Ethics oversight	See above (use of a freely available dataset). Participants gave informed consent, and all recruitment and acquisition methods were approved by the Washington University Institutional Review Board (IRB), following all relevant guidelines and regulations.

Note that full information on the approval of the study protocol must also be provided in the manuscript.

Field-specific reporting

Please select the one below that is the best fit for your research. If you are not sure, read the appropriate sections before making your selection.

☒ Life sciences ☐ Behavioural & social sciences ☐ Ecological, evolutionary & environmental sciences

For a reference copy of the document with all sections, see nature.com/documents/nr-reporting-summary-flat.pdf

Life sciences study design

All studies must disclose on these points even when the disclosure is negative.

Sample size	Participants were extracted from the 1200 Subjects Release (S1200) of the Human Connectome Project (HPC) dataset, with the following inclusion criteria: age range: 26-30 years old; MRI field: 3T, DTI data (not analysed for this study). A total of 390 participants (173 males) were included in the study.
Data exclusions	Participants with imaging quality control issues, major neurological diseases, psychiatric or medical disorders were excluded.
Replication	The sulcal patterns were independently classified by two experts (inter-rater reliability of 87% for the left hemisphere and 88% for the right hemisphere). If the two experts disagreed on the classification of a given participant, a third expert joined the other experts to reach a consensus.
Randomization	Only one group was selected for the study. The sample was then divided according to the sulcal pattern of each participant.
Blinding	Visual inspection of the left and right sulcal pattern was performed blind to potentially confounding information, including participant's age and cognitive scores.

Reporting for specific materials, systems and methods

We require information from authors about some types of materials, experimental systems and methods used in many studies. Here, indicate whether each material, system or method listed is relevant to your study. If you are not sure if a list item applies to your research, read the appropriate section before selecting a response.

Materials & experimental systems

n/a	Involved in the study	
☒	☐	Antibodies
☒	☐	Eukaryotic cell lines
☒	☐	Palaeontology and archaeology
☒	☐	Animals and other organisms
☒	☐	Clinical data
☒	☐	Dual use research of concern
☒	☐	Plants

Methods

n/a	Involved in the study	
☒	☐	ChIP-seq
☒	☐	Flow cytometry
☐	☒	MRI-based neuroimaging

Magnetic resonance imaging

Experimental design

Design type	Anatomical-behavioral correlation.
Design specifications	Only structural images were used in the study, both from a morphological and a morphometric point of view.
Behavioral performance measures	The behavioral tasks performed outside the scanner are those described in the freely available Human Connectome Project (HPC) website, 1200 Subjects Release (S1200) at this address: https://db.humanconnectome.org/app/template/Login.vm.

Acquisition

Imaging type(s)	Structural
Field strength	3T
Sequence & imaging parameters	The anatomical MRI corresponded to T1 and T2-weighted images acquired using a 3D Magnetization Prepared Rapid Acquisition Gradient Echo (MPRAGE) sequence (TR = 2400ms; TE = 2.14 ms; TI = 1000 ms; flip angle = 8◦; FOV = 224 x 224 mm; voxel size = 0.7 mm isotropic).
Area of acquisition	Whole brain

Diffusion MRI ☐ Used ☒ Not used

Preprocessing

Preprocessing software	FreeSurfer version 5.2: PreFreeSurfer, FreeSurfer and PostFreeSurfer pipelines.
Normalization	See Glasser et al. 2013
Normalization template	Conte69
Noise and artifact removal	See Glasser et al. 2013
Volume censoring	See Glasser et al. 2013

Statistical modeling & inference

Model type and settings	After the characterization of IPS morphology in each subject, several analyses were conducted to investigate patterns frequency (also comparing with available frequencies in the literature) and relationship with cognition (after cognitive data reduction through Principal Component Analysis).
Effect(s) tested	1) difference in the occurrence of IPS morphological patterns in the left and the right hemispheres; 2) effect of gender and hemisphere on the likelihood of having a specific IPS pattern; 3) association between sIPS ulcal pattern and cognitive performance, with and without cortical thickness as confounding variable.

Specify type of analysis: ☐ Whole brain ☒ ROI-based ☐ Both

Anatomical location(s)	Morphological analysis: intraparietal sulcus (IPS). Morphometric control analysis: superior and inferior parietal lobules.
Statistic type for inference (See Eklund et al. 2016)	1) exact binomial test; 2) Logistic regression; 3) GLM.
Correction	-

Models & analysis

n/a	Involved in the study
☒	☐ Functional and/or effective connectivity
☒	☐ Graph analysis
☒	☐ Multivariate modeling or predictive analysis
